# Retinal toxicity after facial laser epilation

**Published:** 2020

**Authors:** Ioan Alexandru Plăcintă, Rodríguez Angélica De Freitas, Miriam Rahhal-Ortuño, Patricia Udaondo

**Affiliations:** * University and Polytechnic Hospital la Fe, Valencia, Spain

**Keywords:** facial epilation, retinal toxicity, retinal pigment epithelial detachment, subretinal fluid, Nd:YAG, laser epilation

## Abstract

A 39-year-old man presented to the ophthalmology emergency room complaining of decreased visual acuity and metamorphopsia on his left eye after undergoing Nd:YAG facial hair epilation without wearing proper eye protection. Fluorescein angiography showed a foveal staining initially, with an increase in foveal leakage over late phases. On optical coherence tomography, a subfoveal retinal pigment epithelial detachment with associated subretinal fluid and no signs of choroidal neovascularization or cystoid macular edema was observed. Observation with monthly follow-ups was adopted. By the fourth month, the subretinal fluid had already disappeared and visual acuity had returned to 20/20. Over one-year follow-up, the retinal pigment epithelial detachment experienced a decrease in its size. Conservative management may be a valid option in assessing accidental foveal photocoagulation when choroidal neovascularization or cystoid macular edema is absent. The use of wavelength specific goggles is mandatory for the patient and the aesthetician operating the cosmetic laser, especially when operating on the face or around the eyes.

**Abbreviations:** Nd:YAG = neodymium-doped yttrium aluminium garnet, OCT = optical coherence tomography, PED = pigment epithelial detachment, CME = cystoid macular edema

## Introduction

Laser depilation has become extremely popular in the last years, due to the beauty canon change and the cheapening and greater availability of aesthetic lasers. Facial laser depilation is a very common procedure realized in many beauty clinics. The most widely used lasers for aesthetic purposes, deliver energy under a wavelength of 700-1200 nm: alexandrite laser (755 nm), diode laser (800-810 nm), neodymium-doped yttrium aluminium garnet (Nd:YAG) laser (1064 nm) and intense pulsed light lasers (590-1200 nm) [**1**]. When not complying with safety measures, the eyes may be harmed during the laser operation.

## Case report

A 39-year-old man with unremarkable medical and ophthalmological history presented to the ophthalmology emergency room, complaining of metamorphopsia and vision loss on his left eye, after receiving a facial laser hair removal session the week before. The laser used was a 1064 nm Nd:YAG laser. Three days before he was diagnosed with keratoconjuctivitis sicca and treated with ocular lubricants. 

**Fig. 1:**
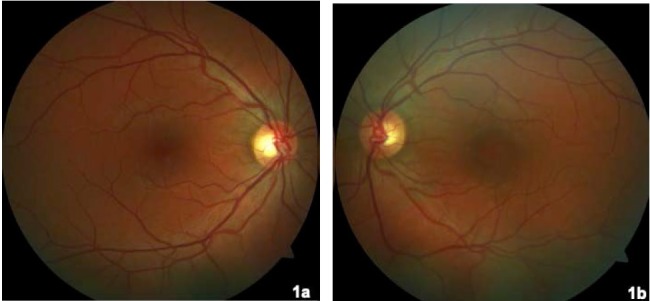
Fundus retinography. **1a.** Right eye fundus retinography with normal foveal glow. **1b.** Left eye fundus retinography with perifoveal hyperpigmentation

**Fig. 2:**
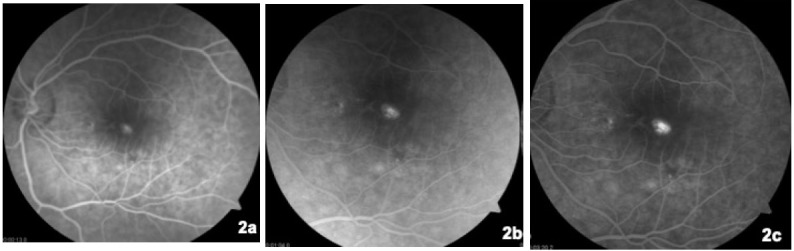
Left eye fluorescein angiography composition. **2a.** Foveal hyperfluorescence 13 seconds after fluorescein injection. **2b.** Increasing leakage at 1 minute after fluorescein injection. **2c.** Further increasing of foveal leakage at 3 minutes and 30 seconds

**Fig. 3:**
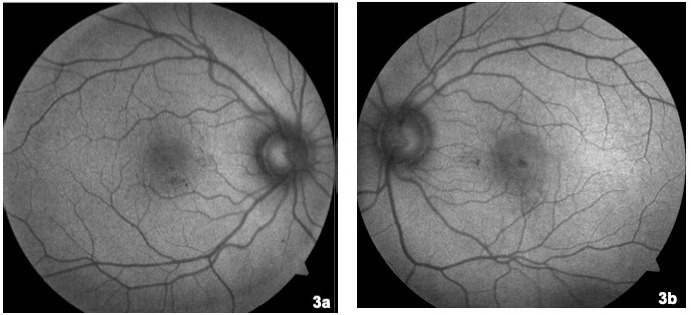
Fundus autofluorescence. **3a.** Right eye depicting normal autofluorescence pattern. **3b.** Left eye. Foveal hypoautofluorescence spot can be observed surrounded by a hypoautofluorescent halo

**Fig. 4:**
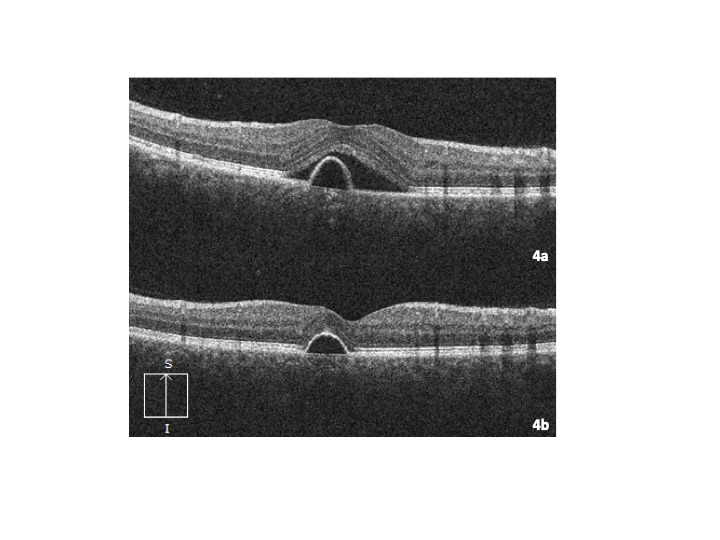
Left eye optical coherence tomography B scan. **4a.** At presentation: retinal epithelium detachment with associated subfoveal serous detachment can be observed. **4b.** 12 months into the follow-up: absence of subretinal fluid with persistence of retinal epithelium detachment

On that occasion, his visual acuity was 20/20 in his right eye and 20/32 in his left eye. No pathological findings on anterior pole slit lamp examination were observed. Intraocular pressure measured by Goldman applanation tonometry was normal (17/18 mmHg). Retinography of his left eye revealed perifoveal hyperpigmentation (**Fig. 1**) compared to the right eye, which had a preserved foveal glow. Fluorescein angiography showed an early hyperfluorescent spot in the foveal area, with increased leakage during the late phases (**Fig. 2**). On autofluorescence, a hypoautofluorescent halo was observed surrounding a hypoautofluorescent foveal spot (**Fig. 3**). Optical coherence tomography (OCT) B scan revealed a subfoveal retinal pigment epithelium detachment (PED) with subretinal fluid; there were no signs of choroidal neovascularization (**Fig. 4a**).

Given our patient’s mild visual acuity loss and absence of choroidal neovascularization, we opted for close follow-ups every 4 weeks, when we measured visual acuity and performed OCT. Visual acuity slowly improved to 20/20 over the first 4 months. At the same time, we observed the vanishing of the subretinal fluid during this period of time. The PED did not display any change at four months. At 12 months, vision remained 20/20 but the patient still complained of a small scotoma and metamorphopsia, mainly in his near vision. On OCT B scan, the PED had experienced a decrease in its size (**Fig. 4b**). 

## Discussion

Cosmetic lasers work under the principle of selective photothermolysis [**1**]; the laser beam penetrates deep enough into the dermis and is mainly absorbed by the target chromophore (the melanin of the hair follicle in the case of laser epilation) inducing a sudden rise of temperature responsible for protein coagulation and tissue necrosis. The beam is pulsed at a fast-enough frequency to produce damage only to the hair follicle without harming the surrounding dermis. 

When using lasers in the periocular area, there is a risk of harming the iris, the retinal pigment epithelium (RPE) or the choroid, as they are chromophore rich tissues able to absorb the energy. Retina hazard range goes form 400 nm up to 1400 nm, correlating with the wavelength spectrum of aesthetic lasers. The eyes are prone to laser injury due the thinner eyelid dermis and Bell’s phenomenon, which exposes the pigmented eye structures to the laser’s range of action.

In a 21 case reports review [**2**] about ocular injuries secondary to face aesthetic laser treatments, the patients were found to experience a broad spectrum of eye globe injuries such as superficial punctate keratitis, iris atrophy, iris transillumination defects, anterior uveitis [**3**], pupillary distortion, posterior synechia or retinal burns [**4**] causing field defects and preretinal hemorrhages. In 62% of the cases, eye protection was not worn at the moment of laser application [**2**]. A literature review regarding the adherence to optical safety guidelines for laser hair depilation found that 72,3% of the cosmetic centers had a lack of non-reflective floors and 59,6% of the providers did not instruct patients not to look into the laser beam or its reflection even while wearing protection goggles [**5**].

The most common way of protecting the eyes is by wearing wavelength-specific spectacles by both patient and the operating cosmetician. Laser-impenetrable metal corneal shields can be fit as contact lenses over the patient’s corneas but they do not exempt the operator of wearing laser goggles. Two case reports of retinal injuries produced by Nd:YAG laser affecting the operating aesthetician have been published so far [**6**,**7**]. In both of them, the operator was either not wearing eye protection at all [**6**] or wearing regular sunglasses [**7**]. 

Our patient was not wearing any kind of ocular protection. The laser beam reached the subfoveal RPE producing its detachment and fluid exudation into the subretinal space. Choroidal neovascularization was not observed in the follow-up but it has been described in the first three weeks after foveal exposure to laser beams. When present, treatment with 1 to 5 doses of intravitreal bevacizumab [**8**] or ranibizumab [**9**] has been used with excellent anatomical and visual outcomes. Cystoid macular edema (CME) has also been described as a complication of macular photocoagulation. When present, treatment with oral, retrobulbar or intravitreal steroids is recommended [**10**]. 

Regarding our patient, we opted for close follow-ups without treatment, given the absence of choroidal neovascularization or CME. Subretinal fluid slowly reabsorbed by itself with recovery of 20/20 visual acuity. Moreover, the RPE detachment experienced a reduction in its size during the one-year follow-up. Fortunately, our patient fully recovered his visual acuity, only complaining of mild scotoma at near vision. Recently, a case of macular burn has been published, following alexandrite laser epilation, leading to permanent scotoma and psychological depression and requiring prescription of antidepressant medication and psychotherapy [**11**].

## Conclusion

There is a risk of accidental foveal photocoagulation associated to the use of cosmetic Nd:YAG laser for facial hair epilation, which increases when safety-operating measures are not followed. Conservative management could be a valid option to assess accidental macular photocoagulation, particularly when there are no choroidal neovascularization or CME associated. The use of wavelength specific goggles by the patient and the operator and the instruction of the patient not to stare into the laser beam or any of its reflections even when wearing eye protection, are mandatory safety measures to follow when utilizing aesthetic lasers in the facial and periocular areas.

**Compliance with ethical standards**

An informed consent was obtained from the patient included in the Case Report.

**Acknowledgments**

None.

**Sources of Founding**

None.

**Disclosures**

None.
